# Dendritic atrophy constricts functional maps in resonance and impedance properties of hippocampal model neurons

**DOI:** 10.3389/fncel.2014.00456

**Published:** 2015-01-12

**Authors:** Neha Dhupia, Rahul K. Rathour, Rishikesh Narayanan

**Affiliations:** ^1^Cellular Neurophysiology Laboratory, Indian Institute of ScienceBangalore, India; ^2^Centre for Converging Technologies, University of RajasthanJaipur, India

**Keywords:** dendritic morphology, functional maps, HCN channel, impedance, resonance

## Abstract

A gradient in the density of hyperpolarization-activated cyclic-nucleotide gated (HCN) channels is necessary for the emergence of several functional maps within hippocampal pyramidal neurons. Here, we systematically analyzed the impact of dendritic atrophy on nine such functional maps, related to input resistance and local/transfer impedance properties, using conductance-based models of hippocampal pyramidal neurons. We introduced progressive dendritic atrophy in a CA1 pyramidal neuron reconstruction through a pruning algorithm, measured all functional maps in each pruned reconstruction, and arrived at functional forms for the dependence of underlying measurements on dendritic length. We found that, across frequencies, atrophied neurons responded with higher efficiency to incoming inputs, and the transfer of signals across the dendritic tree was more effective in an atrophied reconstruction. Importantly, despite the presence of identical HCN-channel density gradients, spatial gradients in input resistance, local/transfer resonance frequencies and impedance profiles were significantly constricted in reconstructions with dendritic atrophy, where these physiological measurements across dendritic locations converged to similar values. These results revealed that, in atrophied dendritic structures, the presence of an ion channel density gradient alone was insufficient to sustain homologous functional maps along the same neuronal topograph. We assessed the biophysical basis for these conclusions and found that this atrophy-induced constriction of functional maps was mediated by an enhanced spatial spread of the influence of an HCN-channel cluster in atrophied trees. These results demonstrated that the influence fields of ion channel conductances need to be localized for channel gradients to express themselves as homologous functional maps, suggesting that ion channel gradients are necessary but not sufficient for the emergence of functional maps within single neurons.

## Introduction

Hippocampal pyramidal neurons exhibit tremendous morphological variability. These variations in morphology could be simple baseline variability (Bannister and Larkman, 1995; Ishizuka et al., 1995; Dougherty et al., 2012), or be a consequence of structural plasticity that is associated with several physiological and pathophysiological conditions (Leuner and Gould, 2010). Specifically, structural plasticity in the CA1 subregion has shown to be associated with development (Pokorny and Yamamoto, 1981), aging (Lolova, 1989; Kadar et al., 1994; Markham et al., 2005; Mora et al., 2007), reproductive experience (Pawluski and Galea, 2006), enriched environment (Faherty et al., 2003; Leggio et al., 2005) and with pathological conditions that include Alzheimer's disease (Hanks and Flood, 1991; De Leon et al., 1997; Elgh et al., 2006; Kerchner et al., 2012), various forms of chronic stress (Lambert et al., 1998; McEwen, 1999; Bartesaghi et al., 2003; Isgor et al., 2004; Brunson et al., 2005; Pinto et al., 2014) and depression (Sheline et al., 1996; Campbell and Macqueen, 2004). What are the functional consequences of such innate and remodeling-dependent changes in pyramidal neuron morphology? Primarily, it has been shown that changes in dendritic arborization could alter synaptic and neuronal excitability, somatodendritic coupling, firing properties and firing patterns (Mainen and Sejnowski, 1996; Krichmar et al., 2002; Van Ooyen et al., 2002; Sjöström et al., 2008; Narayanan and Chattarji, 2010; Torben-Nielsen and Stiefel, 2010; Van Elburg and Van Ooyen, 2010; Ferrante et al., 2013; Platschek et al., 2013), and modulate forward/back-propagation of electrical potentials (Vetter et al., 2001; Sjöström et al., 2008; Narayanan and Chattarji, 2010; Ferrante et al., 2013) and coincidence detection (Schaefer et al., 2003). Although the modulation of these physiological measurements emphasize an important role for neuronal morphology in action potential firing and in the response of a neuron to a single or an array of synaptic inputs, these studies do not address the role of neuronal morphology on frequency-dependent response properties of neurons. Such analyses are critical because hippocampal neurons reside in an oscillating network where inputs arrive at specific frequency bands (Buzsaki, 2002, 2006; Wang, 2010), and their responses are tuned to optimally respond to these frequency bands (Gimbarzevsky et al., 1984; Hutcheon and Yarom, 2000; Pike et al., 2000; Hu et al., 2002; Narayanan and Johnston, 2007). In an effort to fill this lacuna, in this study, we quantitatively assessed the role of neuronal morphology on location- and frequency-dependent response properties of hippocampal pyramidal neurons and their dendrites.

Hippocampal pyramidal neurons typically respond to afferent oscillatory inputs with spikes at specific oscillatory phases of these inputs (O'keefe and Recce, 1993; Buzsaki, 2002, 2006; Wang, 2010). In doing so, they recruit several voltage-gated ion channels (VGIC) in tuning their frequency-dependent response properties in a stratified manner enabling location-dependent input processing. A classic and extremely useful measure of frequency-dependent neuronal responses is electrical impedance, which is simply defined as the ratio of voltage response to the current input at various input frequencies (Cole, 1932, 1968; Cole and Baker, 1941). Impedance measurements from hippocampal pyramidal neurons have shown that they exhibit a maximal response at a particular frequency (known as the resonance frequency) in the theta-frequency range (4–10 Hz), and that the impedance phase is positive (inductive) in this theta-frequency range (Gimbarzevsky et al., 1984; Pike et al., 2000; Hu et al., 2002, 2009; Narayanan and Johnston, 2007, 2008; Vaidya and Johnston, 2013). Several ion channels with specific constraints on their kinetics and voltage-dependent properties could elicit such resonance, and these resonance properties could be dependent on dendritic location based on several mechanisms (Gimbarzevsky et al., 1984; Hutcheon and Yarom, 2000; Pike et al., 2000; Hu et al., 2002, 2009; Narayanan and Johnston, 2007, 2008; Vaidya and Johnston, 2013; Zhuchkova et al., 2013; Laudanski et al., 2014).

In hippocampal pyramidal neurons, impedance-dependent properties have been shown to be location-dependent, with both resonance frequency and inductive phase varying with distance from the cell body. Specifically, two forms of resonance have been reported to express in hippocampal pyramidal neurons, with complementary location-dependent profiles. The hyperpolarization-activated cyclic-nucleotide gated (HCN) channels mediate resonance at more hyperpolarized voltages, and the *M*-type K^+^ channels mediate resonance at relatively depolarized voltages. Furthermore, resonance frequency measured at hyperpolarized voltages increases with distance from the cell body whereas that at depolarized voltage ranges decreases with distance from the cell body, thereby complementing each other both as functions of voltage range as well as somatodendritic location (Pike et al., 2000; Hu et al., 2002, 2009; Narayanan and Johnston, 2007, 2008, 2012).

What mediates these resonance frequency maps in hippocampal pyramidal neurons? Several lines of experimental and modeling evidence suggest that these topographic functional maps are *actively* mediated by ion channel localization profiles. First, HCN channels express at higher densities in the distal dendrites of hippocampal pyramidal neurons (Magee, 1998; Lorincz et al., 2002), *M*-type K^+^ channels are largely perisomatic (Hu et al., 2007). In conjunction with the monotonic relationship between resonating conductance density and resonance frequency (Hutcheon et al., 1996; Narayanan and Johnston, 2007), this suggests that the resonance frequency maps reflect the respective conductance gradient. Second, pharmacological blockade of these channels in hippocampal pyramidal neurons clearly demonstrates that the associated resonance frequency maps are exclusively dependent on the specific ion channels. Specifically, at hyperpolarized voltages, blockade of HCN channels rendered the somatodendritic structure to be simple low-pass structures with the abolishment of resonance and phase lead in the voltage response, across dendritic locations (Narayanan and Johnston, 2007, 2008; Hu et al., 2009; Vaidya and Johnston, 2013). On the other hand, at depolarized voltages, blocking *M*-type K^+^ channels eliminated band-pass characteristics of somatodendritic response properties (Hu et al., 2002, 2007). Third, if resonance were merely reflective of passive gradients in the neuronal topograph, maintaining two spatial gradients with opposing signs would be infeasible. However, in a hippocampal pyramidal neuron, two distinct complementary location-dependent gradients of resonance and impedance profiles express on the *same* neuronal topograph and reflect the corresponding channel localization profiles. Fourth, and importantly, input resistance and impedance properties are rendered largely location-independent when HCN channels are blocked (Narayanan and Johnston, 2007, 2008), implying that resistance and impedance gradients at hyperpolarized voltages express only in the presence of HCN channels. Therefore, it is imperative that the presence of a gradient in HCN channels is essential in mediating these *coexisting* functional maps of input resistance and local/transfer impedance properties (Narayanan and Johnston, 2007, 2008, 2012; Vaidya and Johnston, 2013). Finally, modeling studies performed in the presence of these important experimental constraints have clearly demonstrated that a constant density of HCN channels or a shallow gradient in their density is insufficient to elicit these coexistent maps of input resistance and local/transfer resonance properties (Hu et al., 2002, 2009; Narayanan and Johnston, 2007, 2008; Vaidya and Johnston, 2013).

Together, these experimental and modeling studies on hippocampal pyramidal neurons demonstrate that a somatodendritic gradient in the density of a resonating conductance is *necessary* for the expression of the functional maps in input resistance, resonance frequency and other impedance properties (Narayanan and Johnston, 2007, 2008, 2012). In other words, in a hippocampal pyramidal neuron, in the absence of the gradient in the density of the resonating conductance, the specific functional map ceases to exist. Is a gradient in the density of a resonating conductance *sufficient* to impose these functional maps on a neuronal topograph? What is the impact of dendritic structure on how neurons respond to time-varying inputs under conditions where the neuron is passive? How does such impact change when the same neurons express gradients in ion channel properties/densities? Do functional maps of impedance-related and resonance measurements depend on dendritic arborization? To answer these questions, and motivated to assess the role of dendritic morphology on neuronal intrinsic response dynamics, we analyzed several well-established functional maps mediated by HCN channels in conductance-based neuronal models with different morphological complexities.

We found that, in the passive structure or in a neuronal model endowed with a HCN-channel gradient, dendritic atrophy induced a frequency-nonspecific increase in neuronal excitability across dendritic locations and significantly enhanced somatodendritic coupling. Further, assessing local and transfer resonance frequencies in a model endowed with an HCN-channel gradient, we found that the presence of this gradient was not sufficient to impose a map in resonance frequencies in an atrophied neuron. This conclusion was consistent across several other measurements, suggesting that ion channel gradients along a dendritic topograph alone are insufficient to introduce gradients in physiological measurements along the same topograph. Finally, we explored the biophysical basis for these conclusions, and found that our results were a direct consequence of an atrophy-induced increase in the spatial influence of a local channel cluster on several physiological measurements. Our results have important implications for structure-function relationships, especially with reference to neuronal excitability, induction of synaptic plasticity, rate and temporal coding of place fields, channelostasis and targeting of specific ion channels, propagation of electrical and biochemical signals along the dendritic arbor and neuronal spike initiation dynamics, both from physiological and pathophysiological standpoints.

## Materials and methods

**Figure 1:**
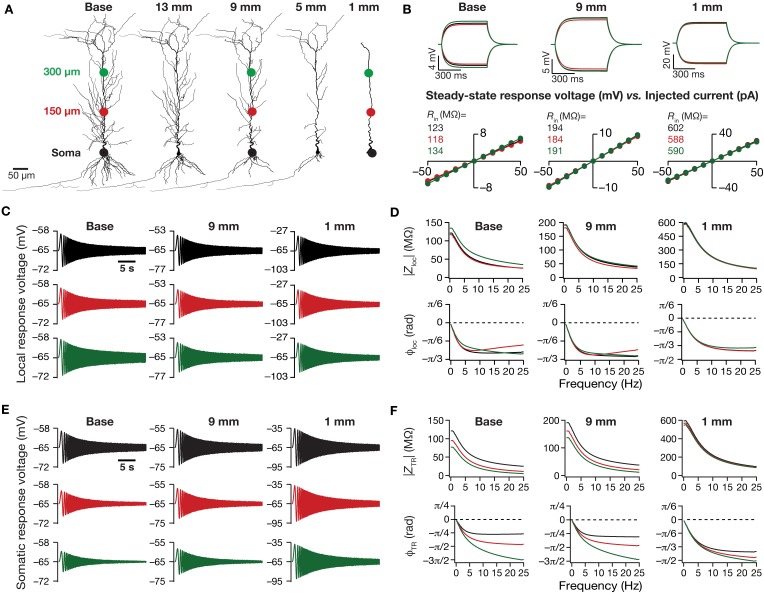
**Traces and measurements related to neuronal intrinsic response dynamics revealed significant somatodendritic changes with dendritic atrophy in a passive neuronal model. (A)** Baseline and a subset of pruned morphologies employed in this study. Base: two-dimensional projection of a CA1 pyramidal neuron reconstruction. The total dendritic length of the neuron was 17.5, 13, 9, 5, and 1 mm are the labels provided for atrophied dendritic reconstruction, and correspond to the total dendritic length of these pruned morphologies. The three colored dots along the trunk marked at the soma (black), and at ~150 μm (red) and ~300 μm (green) away from soma depict the color-coded locations corresponding to the traces and plots shown across all figures of the manuscript. The markings are shown only on dendritic trees where traces/analyses are elaborated in **(B–F)**. **(B)** Local voltage traces (top) and steady-state *V*–*I* plots (bottom) fitted with straight lines obtained in response of depolarizing and hyperpolarizing current pulses injected at three identical locations on three different morphological reconstructions. **(C)** Local voltage traces obtained in response to a chirp stimulus (a sinusoidal current wave of constant amplitude with frequency linearly increasing from 0 to 25 Hz in 25 s) injected at three identical locations on three different morphological reconstructions. **(D)** Local impedance amplitude (top) and phase (bottom) profiles obtained from traces shown in **(C)** and plotted as functions of frequency for various somatodendritic locations and levels of atrophy. **(E)** Somatic voltage traces obtained in response of a chirp stimulus injected at three identical locations on three different morphological reconstructions. **(F)** Transfer impedance amplitude (top) and phase (bottom) profiles obtained from traces shown in **(E)** and plotted as functions of frequency for various somatodendritic locations and levels of atrophy.

In this study, we employed conductance-based multicompartmental models built upon a three-dimensional reconstruction of a hippocampal CA1 pyramidal neuron for understanding the impact of dendritic remodeling on neuronal impedance properties. Progressive dendritic atrophy of this reconstruction was achieved through a pruning algorithm designed to elicit uniform pruning across all dendritic *strata* (Narayanan et al., 2005). The pruning algorithm was employed to create 16 different pruned morphologies, with the difference between successive morphologies set at around 1 mm atrophy of total dendritic length (see Figure 1A for representative examples of pruned morphologies). In what follows, the unpruned morphology will be referred to as the base model, and the other models will be referred by their total dendritic length in mm (Figure 1A). We employed this approach of systematically altering a single base dendritic morphological structure for our study owing to its advantages in comparison to a correlative approach of using different morphological structures and obtaining physiological measurements from them (Narayanan et al., 2005; Narayanan and Chattarji, 2010):

1. The algorithm allows us to induce specific structural changes in a given neuron and examine its functional consequences in the same neuron, thereby enabling us to establish a *causal* link between dendritic remodeling and its biophysical effects.
2. The use of multiple neurons to arrive at the relationship between structure and function of neurons has the potential pitfall that biologically observed statistical variability across neurons might cause a non-atrophied neuron to elicit functional responses similar to an atrophied neuron. Our algorithm uses a single neuron to causally construct the structure–function relationship, thereby precluding the effects of intrinsic variability across morphologies from specific dendritic remodeling.
3. The algorithm provides us with trees with varying percentages of atrophy of the original dendritic tree. This enables us to analyze the functional form of the relationship between various biophysical parameters and the total dendritic length.
4. Comparison of the neuronal responses to stimulation with multiple frequencies at the *same* dendritic point in different trees is made possible because atrophied trees are subtrees of the base reconstruction. This implies that the branching structure remains the same, thereby ensuring that the analysis is not confounded by the impact of branching patterns on the propagation of information along the dendritic tree (Vetter et al., 2001; Ferrante et al., 2013). This is especially necessary in our analysis of local and transfer impedance properties with varying gradients in passive and active properties, where maintaining the same location across trees becomes crucial.

### Multicompartmental model: passive properties

A three-dimensional reconstruction of a hippocampal CA1 pyramidal neuron (*n123*), obtained from NeuroMorpho.Org (Ascoli et al., 2007), originally reconstructed by Pyapali et al. (1998) was used as the base morphology for all multicompartmental simulations. Passive electrical parameters were tuned in a manner such that the local input resistance (*R*_in_) remained constant (~120 MΩ) throughout the trunk (Narayanan and Johnston, 2007). The specific membrane capacitance was set as 1 μF/cm^2^. Specific membrane resistivity *R_m_* and intracellular resistivity *R_a_*, for compartments along the somatodendritic compartments as functions of radial distance from the soma, *x*, were set as:

(1) $$R_{m}(x)=R_{m}^{som}+\frac{\left(R_{m}^{end}-R_{m}^{som}\right)}{1+\exp\left(\frac{250-x}{50}\right)}\text{k}\Omega .{\text{cm}}^{2}$$

(2) $$R_{a}(x)=R_{a}^{som}+\frac{\left(R_{a}^{end}-R_{a}^{som}\right)}{1+\exp\left(\frac{250-x}{50}\right)}\Omega .\text{cm}$$

where *R^som^_m_* = 55 kΩ.cm^2^ and *R^som^_a_* = 70 Ω.cm were the values at soma, *R^end^_m_* = 20 kΩ.cm^2^ and *R^end^_a_* = 30 Ω.cm were values at distal end of the apical trunk (which was ~450 μm distance from the soma for the reconstruction). The basal dendrites have similar *R_m_* and *R_a_* as somatic compartments. This model was compartmentalized using the d_λ_ rule (Carnevale and Hines, 2006), ensuring that each compartment was smaller than 0.1λ_100_, where λ_100_ constitutes the space constant computed at 100 Hz.

### Channel kinetics and distribution

The kinetics and voltage-dependent properties of the hyperpolarization activated cyclic nucleotide gated (HCN or simply *h*) channel was derived from Magee (1998) and Poolos et al. (2002). In simulations where an *h*-channel gradient was included, the maximal conductance value for the *h* conductance for compartments all over somato-apical arbor, as a function of radial distance from soma, *x* was set as:

(3) $${\bar{g}}_{\text{h}}(x)=50\left(1+\frac{25}{1+\exp\left(-(x-350)/15\right)}\right)\mu {\text{S/cm}}^{2}$$

The basal dendrites had the same g_h_ as the somatic compartments. The values in Equation (3) were tuned in a manner such that the *R*_in_ reduced from ~75 to 40 MΩ along the somatoapical trunk of the base model, with a corresponding increase in resonance frequency (*f_R_*) from 3 to 11 Hz, measured at −65 mV (Narayanan and Johnston, 2007).

### Measurements

All physiological relevant measurements were computed employing procedures listed in previous studies (Narayanan and Johnston, 2007, 2008; Rathour and Narayanan, 2012a, 2014; Das and Narayanan, 2014). Specifially, *R*_in_ was measured as the slope of the *V–I* plot, with *V* representing the local steady-state voltage response to depolarizing and hyperpolarizing current pulses of amplitude *I*, ranging from −50 pA to 50 pA, in steps of 10 pA, for 300 ms, at specific locations along the somatodendritic arbor. In certain cases, to minimize the overall voltage deflections, the current range was reduced to −25 pA to 25 pA in steps of 5 pA.

The stimulus used for computing the impedance was a chirp stimulus, a sinusoidal current wave with constant amplitude (50 pA), with frequency linearly increasing from 0 to 25 Hz in 25 s. For models endowed with HCN channels, the amplitude of the chirp stimulus waveform was normalized with respect to input resistance of somatic compartment such that peak-to-peak amplitude of voltage response was similar across various pruned morphologies. Two types of impedance measurements were performed: local and transfer. For local measurements, the voltage response was recorded at the same location where the chirp stimulus was injected. For transfer impedance measurements, on the other hand, the chirp stimulus was injected at different somatodendritic locations, but the voltage response was always recorded at the soma. *Z_loc_*(*f*) and *Z_TR_*(*f*) were used to represent local and transfer impedance, respectively. When impedance properties were represented as functions of distance, the distance corresponded to the location of chirp injection.

The magnitude of the ratio of the Fourier transform of voltage response to the Fourier transform of the chirp stimulus formed the impedance amplitude profile (ZAP). The impedance magnitude for a given impedance *Z*(*f*) was calculated using following equation:

(4) $$\left|Z(f)\right|=\sqrt{{\left(Re(Z(f))\right)}^{2}+{\left(Im(Z(f))\right)}^{2}}$$

where Im(*Z*(*f*)) and Re(*Z*(*f*)) were the imaginary and real parts of the impedance *Z*(*f*), respectively.

The frequency at which the *Z*_loc_(*f*) and *Z*_TR_(*f*) reached their maximum was considered as the local (*f*_R_) and transfer (*f*_TR_) resonance frequency, respectively (Hutcheon and Yarom, 2000; Hu et al., 2002, 2009; Narayanan and Johnston, 2007; Vaidya and Johnston, 2013). |*Z_loc_*|_max_ and |*Z*_TR_|_max_ represented the maximum values of the local and transfer ZAP, which as per definition equal |*Z*_loc_(*f*_R_)| and |*Z*_TR_(*f*_TR_)|, respectively. Local resonance strength (*Q*) was measured as the ratio of |*Z*_loc_(*f*_R_)| to |*Z*_loc_(0.5)| and transfer resonance strength (*Q*_TR_) was measured as the ratio of |*Z*_TR_(*f*_TR_)| to |*Z*_TR_(0.5)| (Hu et al., 2002; Das and Narayanan, 2014).

Impedance phase profile (ZPP) for a given impedance *Z*(*f*) was calculated as:

(5) $$\phi (f)={\text{tan}}^{-1}\frac{Im\left(Z(f)\right)}{Re\left(Z(f)\right)}$$

where ϕ_loc_(*f*) and *ϕ*_TR_(*f*), computed respectively from *Z*_loc_(*f*) and *Z*_TR_(*f*) using Equation (5), represented local and transfer ZPPs, respectively. The total inductive phase was computed as the area under the inductive part of the corresponding ZPP (Narayanan and Johnston, 2008; Rathour and Narayanan, 2014):

(6) $$\Phi_{L}(f)=\int_{\phi (f)\;>\;0}\phi (f)df$$

Φ*_L_*(*f*) and Φ^TR^_*L*_(*f*), computed respectively from *ϕ*_loc_ (*f*) and *ϕ*_TR_(*f*) using Equation (6), represented the total inductive phase for local and transfer ZPPs, respectively.

### Influence field quantification

The influence field for an ion channel cluster inserted at location *x_i_*, for measurement *M*, was calculated through the normalized influence factor, Λ*_M_* (*x*;*x_i_*) as follows (Rathour and Narayanan, 2012b):

(7) $$\Lambda_{M}\left(x;x_{i}\right)=\frac{{\text{IF}}_{\text{M}}(x;x_{i})}{\max{\text{IF}}_{\text{M}}(x;x_{i})}$$

where *x* stands for location along trunk length and IF_M_ (*x; x_i_*) is the unnormalized influence factor, calculated as:

(8) $${\text{IF}}_{\text{M}}(x;x_{i})=\frac{\left|M_{\text{org}}(x)-M_{\text{new}}(x;x_{i})\right|}{M_{\text{org}}(x)}$$

where *M*_org_ represented the measurement obtained in absence of the ion channel cluster at location *x_i_* and *M*_new_ was obtained after inserting channel cluster at location *x_i_*. For example, for measuring the influence field of an HCN-channel cluster on *R*_in_, *R*_org_ (*x*) and *R*_new_ (*x; x_i_*) were calculated in the absence and in the presence of the channel cluster located at *x_i_*, respectively. Unless otherwise stated, the HCN-channel cluster was located at a dendritic path distance of *x_i_* = ~450 μm away from soma (around the center of the apical trunk).

For quantitative analyses of the influence field, we employed the area under the influence field plot as a measure of the extent of influence of a single ion channel cluster located at *x_i_*:

(9) $$\text{AUC of }\Lambda_{M}=\int_{0}^{L_{d}}\Lambda_{M}(x;x_{i})dx$$

where *L_d_* is the total path length of the apical trunk. Whereas Λ*_M_* (*x; x_i_*) was employed for computing the area under the curve (AUC) of the normalized influence field, for computing the AUC of the unnormalized influence field, we employed IF*_M_*(*x; x_i_*) (Equation 8).

### Computational details

All simulations were performed using the NEURON simulation environment (Carnevale and Hines, 2006). Simulations were performed with the membrane potential set at −65 mV. The temperature was set at 34°C, and ion channel kinetics were adjusted appropriately to account for their experimentally determined *Q*_10_ factors. The default integration time step for the simulations was set at 25 μs. Computation of physiologically-relevant measurements from simulation traces and quantification of influence fields were performed with custom-built software written within the IGOR Pro (Wavemetrics) programming environment.

## Results

How does dendritic morphology alter intrinsic response dynamics in a passive neuronal model? To address this question, we employed a 3D reconstructed CA1 pyramidal neuron as our base morphology and adjusted its passive properties to match with experimental measurements (Narayanan and Johnston, 2007). We then applied an iterative pruning algorithm (Narayanan et al., 2005) on this base morphology to obtain 17 different morphologies, each successively pruned by ~1 mm (from the ~17.5 mm of total dendritic length of the base morphology to ~1 mm of total dendritic length of the most pruned morphology; Figure 1A). We employed these pruned reconstructions, imposed identical passive properties on each of them and analyzed the impact of dendritic atrophy on somatodendritic excitability in passive neuronal models.

### Dendritic atrophy increased local and transfer impedance amplitudes across locations

**Figure 2:**
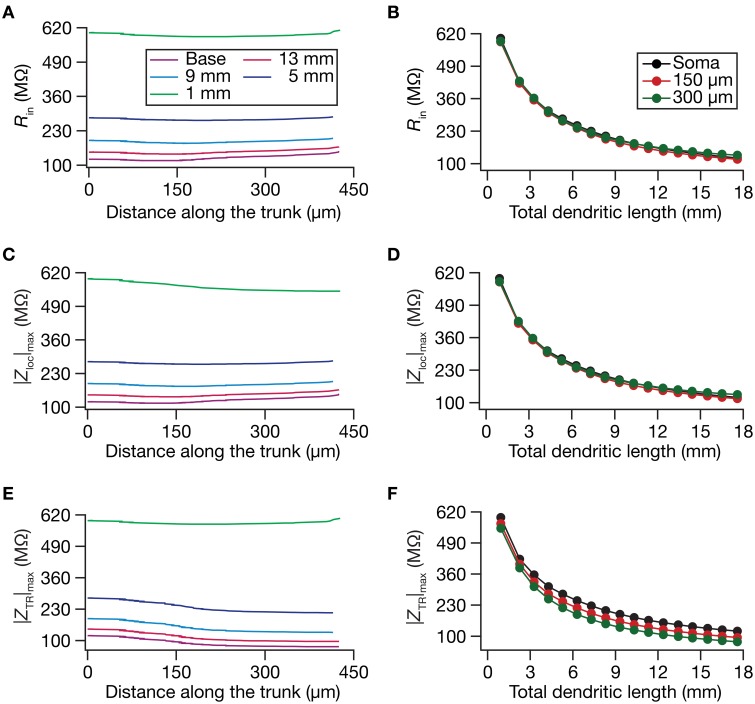
**Steady-state and frequency-dependent measures of excitability increased with dendritic atrophy across somatodendritic locations of a passive neuronal model. (A,B)** Input resistance (*R*_in_) plotted as functions of somatodendritic location (**A**; for 5 different pruned morphologies, Figure 1A) and total dendritic length (**B**; for 3 distinct somatodendritic locations, Figure 1A). **(C,D)** Maximal local impedance amplitude (|*Z*_loc_|_max_) plotted as functions of somatodendritic location **(C)** and dendritic length **(D)**. **(E,F)** Maximal transfer impedance amplitude (|*Z*_TR_|_max_) plotted as functions of somatodendritic location **(E)** and total dendritic length **(F)**. The legends for all graphs on the left and right are given in **(A,B)**, respectively.

We computed *R*_in_ at different locations along the dendritic arbor (Figure 1B), and found that *R*_in_ increased across the dendritic arbor with atrophy (Figures 2A,B). This should be expected because a reduction in total surface area and branching directly translates into an increase in the input resistance of the compartment (Rall, 1977; Mainen and Sejnowski, 1996; Krichmar et al., 2002; Van Ooyen et al., 2002; Narayanan and Chattarji, 2010; Van Elburg and Van Ooyen, 2010). Whereas this steady-state neuronal response property showed an expected outcome, how does the frequency-dependent response of a neuron vary with dendritic remodeling? To address this question, we computed the local impedance amplitude and phase using the chirp stimulus (Figures 1C,D). As our model contained only passive components, the voltage response to a chirp stimulus behaved like a low pass filter, with higher responses at lower frequencies and lower responses at higher frequencies (Figure 1D). Dendritic atrophy did not significantly alter the shape of this low-pass filter (Figure 1D), but changed only the actual response amplitude (Figure 1D). The impedance phase profile always stayed negative across all frequencies (Figure 1D), suggesting that the voltage response lagged the current input at all measured frequencies. This should be expected in a passive system, which behaves similar to an RC circuit, thereby eliciting only negative phases (Cole, 1932, 1968; Cole and Baker, 1941; Narayanan and Johnston, 2008). The shape of the phase response also did not show any significant change with dendritic atrophy across the three measured locations (Figure 1D).

Do conclusions on atrophy-induced increases in the maximal local impedance amplitude at these three locations extend to other locations of the dendritic tree? To address this, we measured |*Z*_loc_|_max_ associated with the local impedance of various locations along the somatoapical trunk, and plotted it for various levels of dendritic atrophy. We found that the maximal local impedance amplitude, measured at several locations along the somatoapical trunk, increased with atrophy (Figures 2C,D).

Information arriving at a dendritic location undergoes a two-step filtering process before integration occurs at the soma. The first is governed by the local frequency response of the dendritic branch, and the second depends on the propagation of this signal along the dendritic tree. A quantitative manner to study the latter is through transfer impedance properties, which may be evaluated by recording the voltage response to a chirp current injected at a somatodendritic location (Ulrich, 2002; Hu et al., 2009; Vaidya and Johnston, 2013). For passive neurons, this transfer impedance profile is expected to be low-pass in nature given the resistor-capacitor electrical structure of a passive neuron (Figures 1E,F). Although the passive nature of the transfer impedance amplitude and phase profiles did not change with dendritic atrophy, atrophy introduced quantitative differences in these profiles (Figure 1F). To quantify this, we computed the transfer impedance amplitude, |*Z*_TR_|_max_, at various locations along the somatoapical trunk, and found that |*Z*_TR_|_max_ shifted to higher values with dendritic atrophy across the entire somatoapical trunk (Figures 2E,F). The pattern of evolution of the transfer impedance amplitude was very similar to the evolution of local impedance amplitude, with a monotonic increase with dendritic atrophy (Figures 2C–F). These, together, implied that atrophied neurons responded with higher efficiency to incoming inputs, and that the transfer of signals across the dendritic tree was more effective in an atrophied tree across frequencies.

### Dendritic atrophy constricted HCN-channel mediated spatial maps of local and transfer impedances

**Figure 3:**
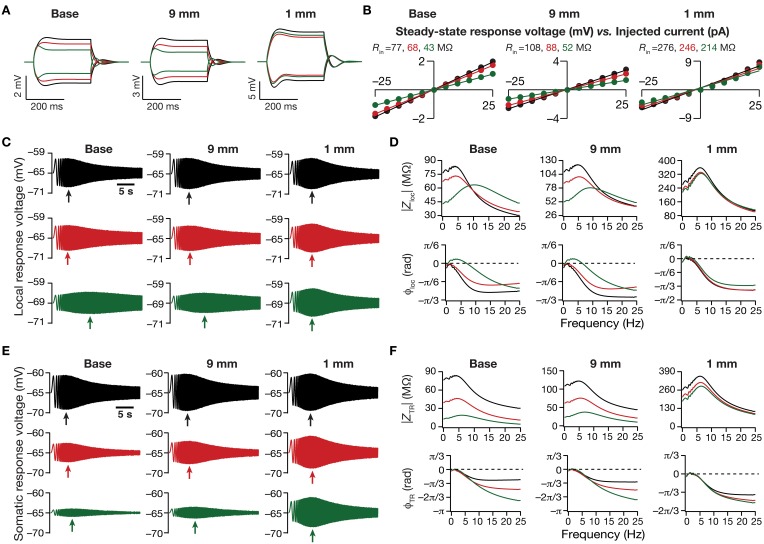
**Dendritic atrophy in the presence of a physiologically constrained HCN-channel gradient introduced significant somatodendritic changes in neuronal intrinsic response dynamics. (A,B)** Local voltage traces **(A)** and steady-state *V*–*I* plots **(B)** fitted with straight lines obtained in response of depolarizing and hyperpolarizing current pulses injected at three identical locations on three different morphological reconstructions. **(C)** Local voltage traces obtained in response of a chirp stimulus injected at three identical locations on three different morphological reconstructions. **(D)** Local impedance amplitude (top) and phase (bottom) profiles obtained from traces shown in **(C)** and plotted as functions of frequency for various somatodendritic locations and levels of atrophy. **(E)** Somatic voltage traces obtained in response of a chirp stimulus injected at three identical locations on three different morphological reconstructions. **(F)** Transfer impedance amplitude (top) and phase (bottom) profiles obtained from traces shown in **(E)** and plotted as functions of frequency for various somatodendritic locations and levels of atrophy. Arrows in **(C,E)** refer to the location of maximal response on those specific traces. See Figure 1A for the morphologies and locations referred here.

**Figure 4:**
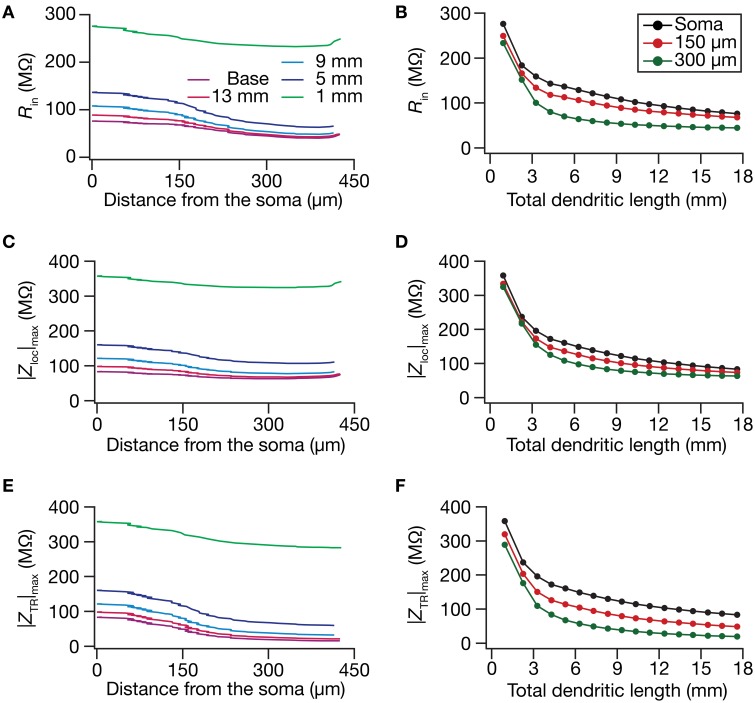
**In the presence of a somatodendritic HCN-channel gradient, steady-state and frequency-dependent measures of excitability increased with dendritic atrophy across somatodendritic locations. (A,B)** Input resistance (*R*_in_) plotted as functions of somatodendritic location (**A**; for 5 different pruned morphologies, Figure 1A) and total dendritic length (**B**; for 3 distinct somatodendritic locations, Figure 1A). **(C,D)** Maximal local impedance amplitude (|*Z*_loc_|_max_) plotted as functions of somatodendritic location **(C)** and total dendritic length **(D)**. **(E,F)** Maximal transfer impedance amplitude (|*Z*_TR_|_max_) plotted as functions of somatodendritic location **(E)** and dendritic length **(F)**. The legends for all graphs on the left and right are given in **(A,B)**, respectively.

Although the input resistance values are flat with low-pass frequency response profiles in the absence of HCN channels, native hippocampal dendrites are endowed with a HCN-channel gradient that mediates a functional gradient of input resistance, and bestows band-pass characteristics on the location-dependent impedance profiles (Magee, 1998; Lorincz et al., 2002; Narayanan and Johnston, 2007; Hu et al., 2009; Vaidya and Johnston, 2013). In hippocampal pyramidal neurons, whereas *R*_in_ stays at almost ~120 MΩ throughout the somatoapical trunk in the absence of the HCN channels, in their presence, *R*_in_ reduces from around 70 MΩ at the soma to around 20–30 MΩ at the distal dendritic locations (Narayanan and Johnston, 2007). To understand the impact of dendritic remodeling on *R*_in_ in the presence of HCN channels, we introduced a density gradient of HCN channels into our morphologies. We imposed the same HCN gradient and passive properties on all the 17 morphologies, and asked if this input resistance map altered with dendritic atrophy. Consistent with our results with passive models (Figures 1, 2), we found that *R*_in_ increased with atrophy (Figures 3A,B). Importantly, we also found that the *R*_in_ map became more uniform in an atrophied neuron, compared to the base morphology (Figures 4A,B). Specifially, whereas there was a reduction in *R_in_* with distance in the base morphology, with atrophy, the ratio between somatic and dendritic *R*_in_ values was lesser in the pruned dendritic tree compared to its control counterpart (Figures 4A,B). Therefore, in atrophied dendritic trees, the gradient in HCN channels was insufficient to maintain a gradient in *R*_in_.

Next, we turned our attention to intrinsic response dynamics associated with time-varying inputs and assessed the impact of dendritic atrophy on impedance profiles in the presence of a HCN channel gradient. In the absence of an inductive element, the local and the transfer impedance profiles are low pass in nature and reflect an RC circuit. However, in the presence of HCN channels, which act as inductive elements, the local (Figures 3C,D) and the transfer (Figures 3E,F) filters resemble band-pass structures with the resonance frequency in the theta frequency range (Hutcheon and Yarom, 2000; Hu et al., 2002, 2009; Ulrich, 2002; Narayanan and Johnston, 2007; Rathour and Narayanan, 2012a; Vaidya and Johnston, 2013). Consistent with the inductive role for HCN channels and their higher densities at distal locations, we also noted that the local impedance phase profile showed significant positive phases for distal dendritic locations, especially in the theta frequency ranges (Figure 3D).

Upon dendritic atrophy, although the band-pass structures of the local and transfer impedance profiles were retained, there were signficant quantitative differences in the amplitude of the responses and their gradients along the somatodendritic axis. Specifically, dendritic atrophy translated into a monotonic increase in local as well as transfer impedance amplitudes (Figures 3C–F, 4C–F). Whereas the atrophy-induced increase in local impedance amplitude could be attributed to a reduction in overall surface area of the neuron, the corresponding increase in transfer impedance amplitude is a direct consequence of the improved somatodendritic coupling in trees with lesser dendritic length and dendritic branch points. Specifically, in the base tree, owing to distance-dependent dendritic filtering, distal inputs were attenuated more compared to proximal inputs, resulting in a progressively lower values for the transfer impedance amplitude with increase in distance (Figures 3E,F, 4E). However, with dendritic atrophy, the somatodendritic coupling was higher owing to the loss of dendritic branches through which information flow could otherwise have been channeled and resulted in an increase in transfer impedance amplitude (Figures 3E,F, 4E). Furthermore, and similar to our observations with *R*_in_, the ZAP profiles across locations were very similar to each other in the atrophied tree (Figures 3D,F), resulting in an atrophy-induced constriction of the somatoapical maps in both local and transfer impedance amplitudes (Figures 4C–F). We also noted that the location-dependent differences in impedance phase plots were significantly reduced with increasing levels of dendritic atrophy (Figures 3D,F).

Together, consistent with our earlier conclusions with passive trees, these results in the presence of a HCN-channel gradient suggested that atrophied neurons responded with higher efficiency to incoming inputs, and that the transfer of signals across the dendritic tree was more effective in an atrophied arborization across frequencies. Importantly, despite the presence of identical HCN-channel density gradients, spatial gradients in input resistance and in local/transfer impedance amplitudes were significantly diminished in neuronal models with dendritic atrophy.

### The somatodendritic local and transfer resonance frequency maps were constricted by dendritic atrophy

**Figure 5:**
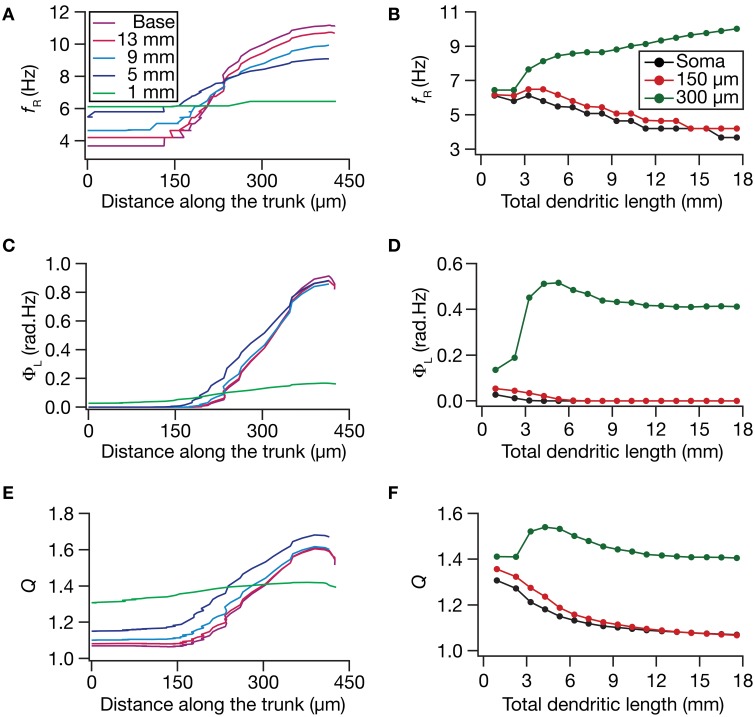
**Functional maps of several HCN-channel-dependent local impedance measurements were critically regulated by dendritic atrophy. (A,B)** Local resonance frequency (*f_R_*) plotted as functions of distance from the soma (**A**; for 5 different pruned morphologies, Figure 1A) and total dendritic length (**B**; for 3 distinct somatodendritic locations, Figure 1A). **(C,D)** Total inductive phase of the local impedance phase profile (Φ_L_) plotted as functions of distance from the soma **(C)** and total dendritic length **(D)**. **(E,F)** Local resonance strength (*Q*) plotted as functions of distance from the soma **(E)** and total dendritic length **(F)**.

How do local and transfer resonance properties and their spatial maps depend on dendritic atrophy? To address this, we first computed the local resonance frequency at several locations along the somatoapical trunk of all pruned reconstructions and plotted it as functions of distance from the soma (Figure 5A) and of dendritic length (Figure 5B). With the insertion of the HCN-channel gradient in the base model, the resonance frequency along the somatodendritic trunk compartments increased ~3-fold with distance from the soma (Figure 5A; Base model), in a manner that was consistent with experimental observations (Narayanan and Johnston, 2007). With atrophy, however, this topographic map of resonance frequency was severely constricted, whereby the distal and proximal resonance frequency values became progressively similar with increasing levels of atrophy (Figures 5A,B). These results suggest that the mere presence of a gradient in HCN channels is insufficient to sustain the resonance frequency map in neurons with lower dendritic length and lesser branches. Apart from the local resonance frequency map, we also computed the maps for total inductive phase (Figures 5C,D) and resonance strength (Figures 5E,F) and found that these conclusions extended to these measurements as well. In conjunction with our conclusions on the input resistance and local impedance amplitude maps (Figure 4), these results suggested that a gradient in HCN-channel density was insufficient to sustain a gradient in several HCN-channel dependent local physiological measurements in an atrophied tree.

**Figure 6:**
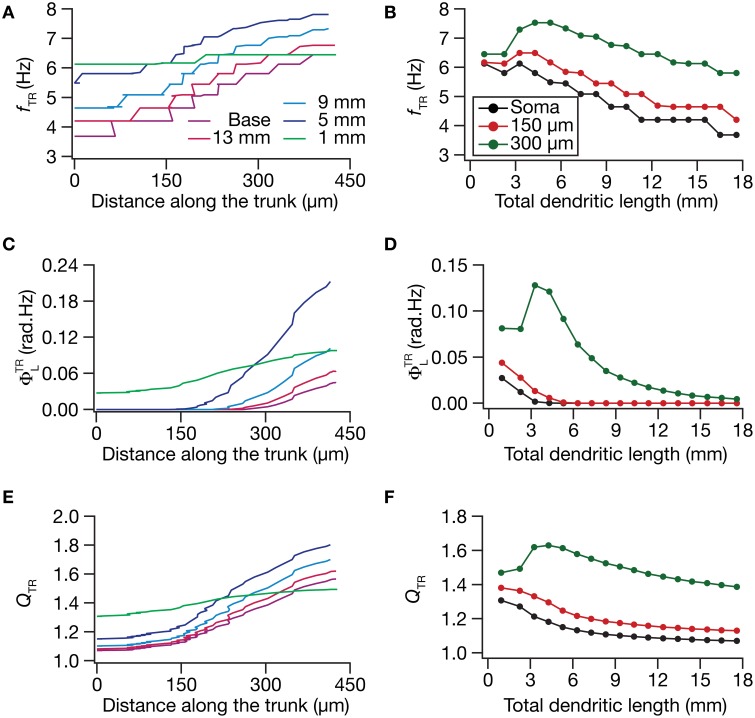
**Functional maps of several HCN-channel-dependent transfer impedance measurements were critically regulated by dendritic atrophy. (A,B)** Transfer resonance frequency (*f_TR_*) plotted as functions of distance from the soma (**A**; for 5 different pruned morphologies, Figure 1A) and total dendritic length (**B**; for 3 distinct somatodendritic locations, Figure 1A). **(C,D)** Total inductive phase of the transfer impedance phase profile (Φ^TR^_L_) plotted as functions of distance from the soma **(C)** and total dendritic length **(D)**. **(E,F)** Transfer resonance strength (*Q_TR_*) plotted as functions of distance from the soma **(E)** and total dendritic length **(F)**.

What was the impact of dendritic atrophy on spectral selectivity in the transfer impedance amplitude profile? To address this, we quantified resonance frequency (Figures 6A,B), total inductive phase (Figures 6C,D), and resonance strength (Figures 6E,F) on transfer impedance profiles computed at different locations on different morphologies. We found that in an atrophied tree, the transfer impedance-related measurements were nearly identical across the somatodendritic axis despite the presence of an underlying HCN-channel gradient. Together these results suggested that the presence of identical HCN-channel density gradient was insufficient to sustain functional maps in input resistance and in local/transfer impedance properties in neuronal models with dendritic atrophy. Specifically, in an atrophied dendritic tree endowed with identical somatodendritic channel gradients as an unatrophied one, several functional maps fail to express and the entire length of the dendrite converges to similar intrinsic response dynamics (Figures 3–6).

### Atrophy-induced constriction of functional maps was mediated by enhanced spatial spread of the influence of a HCN-channel cluster in atrophied trees

Thus far, employing models with a somatodendritic HCN-channel gradient, we had demonstrated that the presence of a somatodendritic gradient in an ion channel density alone was insufficient to establish a functional gradient in a given physiological measurement. What is the biophysical basis for such constricted functional gradients? Why was an identical channel gradient inadequate in sustaining functional maps on atrophied trees? In answering these questions, we reasoned that, in a dendritic tree with heavy arborization, the influence of a point conductance located at any given compartment would be spatially localized owing to the branching and the higher surface area (Williams, 2004; Rathour and Narayanan, 2012b). However, in a dendritic tree with lesser arborization and lower surface area, as a consequence of higher coupling across the compartments (Figures 4E,F), the spread of influence of an ion channel cluster would be enhanced. Together, we hypothesized that this large increase in the influence field of any point conductance would ensure that the impact of this gradient on functional properties is minimized, even in the presence of an ion channel density gradient. In other words, the impact of a dendritically expressed channel is not confined only to the dendritic location, but spreads to a larger extent, thereby altering even somatic properties. This loss of compartmentalization of dendritic conductances, in conjunction with a reciprocally widespread influence of somatic ion channels on dendritic measurements, would ensure that ion channel gradients do not necessarily translate to functional map gradients.

**Figure 7:**
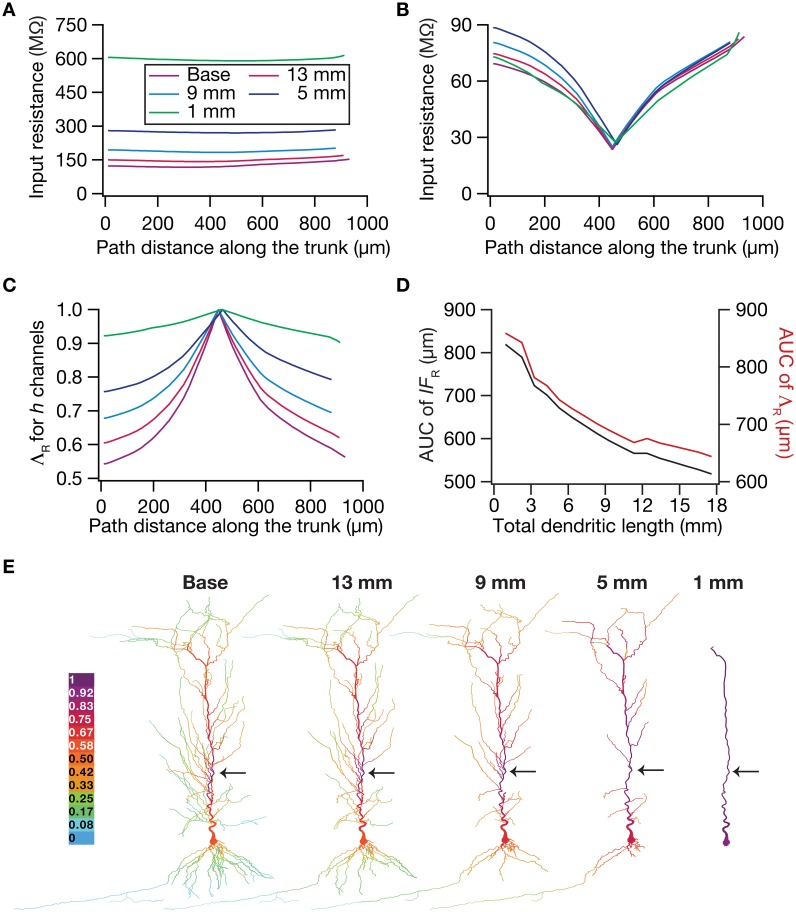
**Dendritic atrophy enhanced the influence of an HCN-conductance cluster on input resistance in a passive model. (A)** Input resistance (*R*_in_) measured along the somato-apical trunk for different morphologies. **(B)** *R*_in_, in different morphologies, measured in the presence of a single HCN-channel cluster, incorporated at ~450 μm (path distance) location away from soma. **(C)** Λ_R_, the normalized influence field for *R_in_*, along the somato-apical trunk for different morphologies. **(D)** Area under curve (AUC) for unnormalized (black) and normalized (red) influence field, plotted as functions of dendritic length of the neuronal morphology under consideration. **(E)** Color-coded influence field across the entire dendritic arbor showing the effect of a single HCN-channel cluster located (black arrow) at ~450 μm from the soma on *R*_in_ for various morphologies.

**Figure 8:**
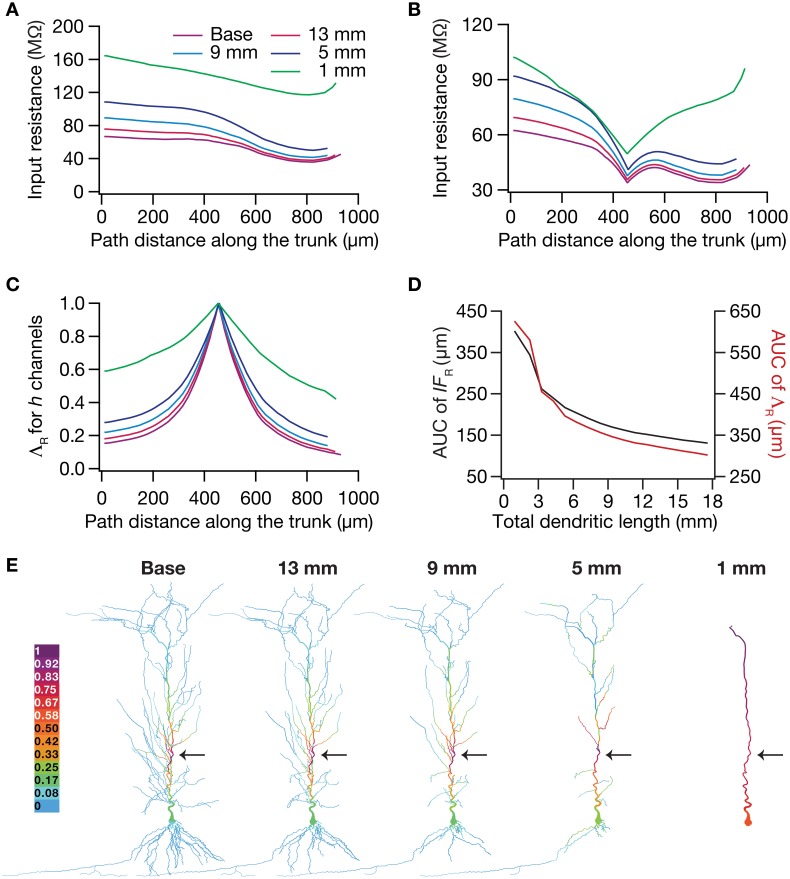
**Dendritic atrophy enhanced the influence of an HCN-conductance cluster on input resistance in a model endowed with a somatodendritic HCN-conductance gradient. (A)** Input resistance measured along the somato-apical trunk for different morphologies. **(B)** *R*_in_, in different morphologies, measured in the presence of an HCN-channel cluster, incorporated at ~450 μm (path distance) location away from soma. **(C)** Λ_R_, the normalized influence field for *R_in_*, along the somato-apical trunk for different morphologies. **(D)** Area under curve (AUC) for unnormalized (black) and normalized (red) influence field, plotted as functions of dendritic length of the neuronal morphology under consideration. **(E)** Color-coded influence field across the entire dendritic arbor showing the effect of a single HCN-channel cluster located (black arrow) at ~450 μm from soma on *R*_in_ for various morphologies.

**Figure 9:**
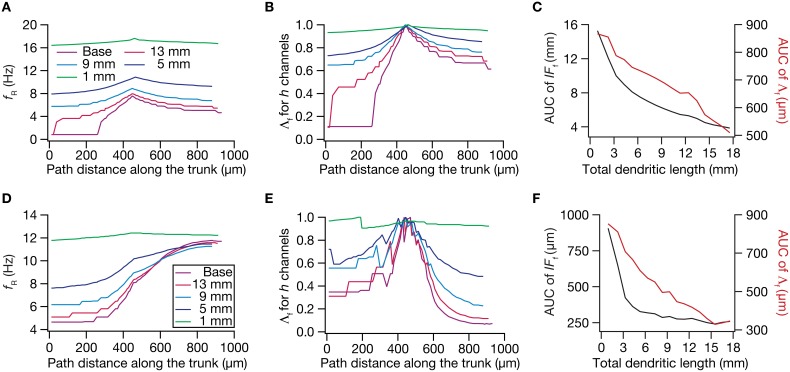
**Dendritic atrophy broadened the influence of a HCN conductance cluster on resonance frequency**. The analyses presented in panels **(A–C)** correspond to passive neuronal models, and those presented in **(D–F)** correspond to models endowed with a physiologically relevant somatodendritic HCN-channel gradient. **(A)** Resonance frequency (*f_R_*), in different morphologies, measured in the presence of an HCN-channel cluster, incorporated at ~450 μm (path distance) location away from soma. **(B)** Λ_f_, the normalized influence field for *f_R_*, along the somato-apical trunk for different morphologies. **(C)** Area under curve (AUC) for unnormalized (black) and normalized (red) influence field, plotted as functions of dendritic length of the neuronal morphology under consideration. **(D)** *f_R_*, in different morphologies, measured in the presence of an additional HCN-channel cluster, incorporated at ~450 μm location away from soma. The baseline values of *f_R_*, in the absence of this additional HCN-channel cluster, were the same as the plots shown in Figure 5A. **(E)** Λ_f_, the normalized influence field of the additional HCN-channel cluster for *f_R_*, along the somato-apical trunk for different morphologies. **(F)** Area under curve (AUC) for unnormalized (black) and normalized (red) influence field of the additional HCN-channel cluster, plotted as functions of dendritic length of the neuronal morphology under consideration.

To test this hypothesis, we employed the recently developed quantification on “influence fields” to assess the spread of influence of a single ion channel cluster on any given measurement (Rathour and Narayanan, 2012b). We picked the two prominent measurements that are sensitive to HCN channels,—input resistance (Figure 4) and resonance frequency (Figure 5)—and asked if the spread of influence of a single HCN-conductance cluster was altered in an atrophied dendritic tree. Specifically, we placed a HCN-conductance cluster (normalized by the specific surface area of the compartment under consideration) at around the center of the somatoapical trunk and quantified the influence field of that cluster (Rathour and Narayanan, 2012b) on input resistance (Figures 7, 8) and resonance frequency (Figure 9). We performed this analysis on either the passive model (Figures 7, 9; where the only active component was this added HCN-conductance cluster) or on the active model (Figures 8, 9; where the baseline HCN-channel gradient was already present, and an additional HCN conductance cluster was appended to it at one location). We employed two different measures to quantify the influence field of the ion channel cluster on the measurements: the area under the curve of the normalized and unnormalized influence fields under these parametric variations.

Employing these two measurements for assessing the influence field of HCN-channel clusters on *R*_in_ and *f_R_* on the 17 different morphologies, each with two different background conductance profiles (passive vs. HCN-channel gradient), we found that the influence field of an ion-channel cluster increased with dendritic atrophy (Figures 7–9). This increase in influence field was not just restricted to the somatoapical trunk (Figures 7–9), but extended to even the obliques (Figures 7E, 8E), and was common for both measurements (Figures 7, 8 for *R*_in_; Figure 9 for *f_R_*) and for both cases of background conductances (Figures 7, 9 for passive and Figures 8, 9 for models with the baseline HCN-channel gradient). These results indicated that the influence fields of ion channel conductances need to be localized for channel gradients to express themselves as functional maps. In the absence of such compartmentalization, such as the case observed in atrophied dendrites, gradients in ion channel properties do not translate into maps in functional properties along the specified neuronal topograph. In other words, HCN-channel gradients are necessary but not sufficient for the emergence of functional maps of input resistance, resonance frequency and impedance properties within single hippocampal pyramidal neurons.

## Discussion

The principal finding of this study is that dendritic morphology critically regulates impedance-related functional maps, with the primary implication that the presence of ion channel gradient alone is not sufficient to impose a continuous gradient of an associated physiological measurement along a neuronal topograph. We arrived at this conclusion using systematically pruned dendritic morphologies, and assessing five local (input resistance, resonance frequency, maximal impedance amplitude, total inductive phase, resonance strength) and four transfer (resonance frequency, maximal impedance amplitude, total inductive phase and resonance strength) measurements on these morphologies. Given that our approach was to prune a specific tree and assess these measurements, we were able to arrive at functional forms for the dependence of each of these measurements on dendritic length. These results clearly show that despite the presence of a gradient in HCN-conductance density along a neuronal topograph, the corresponding physiological measurements do not form a topographic map in atrophied trees. We assessed the biophysical mechanisms behind these observations using the “influence field” framework, and found that these were consequent to an atrophy-induced increase in the spread of influence of an ion channel cluster on these physiological measurements. Apart from these, our study also provides further evidence for a direct relationship between increased excitability and dendritic atrophy, even in frequency-dependent measures of excitability. Further, our results point to an increase in coupling across compartments in an atrophied dendritic tree, ensuring an effective transfer of signals across the somatodendritic axis of an atrophied dendritic tree. These conclusions about morphology-dependent changes in excitability and in functional gradients have direct implications for physiological variability in dendritic length and branching of neurons across different brain regions and for pathophysiological changes in dendritic trees and their branching patterns.

### Implications for atrophy-induced enhancement in neuronal excitability and somatodendritic coupling

The dependence of synaptic and intrinsic neuronal excitability as well as somatodendritic coupling on dendritic arborization is clearly established (Mainen and Sejnowski, 1996; Vetter et al., 2001; Krichmar et al., 2002; Van Ooyen et al., 2002; Schaefer et al., 2003; Kole et al., 2007; Sjöström et al., 2008; Narayanan and Chattarji, 2010; Van Elburg and Van Ooyen, 2010; Ferrante et al., 2013; Platschek et al., 2013). By analyzing local and transfer impedances as functions of input frequency, our results add additional lines of evidence to these conclusions by extending the analyses beyond pulse-current- and firing rate-based measurements of neuronal excitability (Narayanan and Johnston, 2008). Specifically, we show that dendritic atrophy increased both the local and transfer impedance amplitudes across all analyzed frequencies and across all locations along the dendritic arbor. These conclusions also extended to the case where an HCN-channel gradient was present across the somatodendritic gradient, where the local and transfer impedance amplitude profiles were band-pass in structure. These conclusions are especially important in the context of the hippocampus residing in an oscillatory environment where oscillations of different frequencies impinge on the somatodendritic arbor, and mediate various forms of rate, temporal and phase coding (O'keefe and Dostrovsky, 1971; O'keefe and Recce, 1993; Buzsaki, 2002, 2006; Wang, 2010; Lisman and Jensen, 2013). Such widespread increases in frequency-independent excitability, and the tighter somatodendritic coupling inferred from higher transfer impedance amplitudes would together imply that atrophied neurons generate higher number of spikes even for smaller inputs. Further, spike generation in an atrophied dendritic tree would also be expected to be earlier within the theta frequency oscillations. Given this, future studies should focus on the impact of dendritic atrophy on various forms of rate, temporal and phase coding in the hippocampus, including possible expansion in place-cell firing fields and potential saturation in phase precession that is observed in CA1 place cells.

The critical importance of dendritic morphology and surface-area-to-volume ratio in reaction-diffusion systems that regulate biochemical signal transduction (Sabatini et al., 2002; Frick et al., 2003; Neves et al., 2008; Neves and Iyengar, 2009; Kotaleski and Blackwell, 2010; Ross, 2012; Ashhad and Narayanan, 2013) and the vital role that excitability plays in regulating calcium propagation and plasticity rules (Johnston et al., 2003; Schaefer et al., 2003; Sjöström et al., 2008; Narayanan and Johnston, 2010; Ashhad and Narayanan, 2013; Sehgal et al., 2013) are well established. Given these, we postulate that dendritic atrophy and consequent increase in neuron-wide excitability would regulate the amplitudes and propagation of calcium transients, thereby significantly altering the rules for plasticity induction and the spread of signaling components (Narayanan and Chattarji, 2010). If these atrophy-induced changes in excitability were to be nullified for the maintenance of homeostasis in activity, signal propagation and plasticity, then concurrent homeostatic mechanisms should be activated through changes in synaptic and/or intrinsic properties of the neuron (Kole et al., 2004; Turrigiano and Nelson, 2004; Narayanan and Chattarji, 2010; Turrigiano, 2011; Honnuraiah and Narayanan, 2013). Therefore, future studies should recognize morphology as an important additional variable for neurons to adjust local and global neuronal excitability and coupling strengths across compartments, and assess its roles in either maintaining or hampering homeostasis of several neuronal functions.

### Implications for the regulation of functional maps by dendritic atrophy

Our study clearly elucidates the critical role of neuronal morphology in the emergence of several functional maps in input resistance and in local/transfer impedance properties. From the perspective of intraneuronal maps (Narayanan and Johnston, 2012), it should be noted that the maps of local EPSP amplitude and backpropagating action potentials (bAP) have already been shown to be dependent on dendritic remodeling (Vetter et al., 2001; Narayanan and Chattarji, 2010). Together with this, our study establishes that dendritic atrophy plays a significant role in the emergence of functional maps, especially constricting several of these maps despite the presence of ion channel gradients. Physiologically, this implies that the distance-dependent processing capabilities that are enabled by the presence of ion channel gradients would cease to exist under dendritic atrophy or in neurons with severely limited branching profiles. Specifically, the presence of channel gradients introduce location-dependent processing capabilities that regulate the location dependence of spike initiation dynamics, bAP amplitude coincidence detection and frequency-dependent input processing, apart from normalizing temporal summation and input phase of the transfer impedance profiles (Magee, 1998, 1999, 2000; Hausser et al., 2000; Vetter et al., 2001; Schaefer et al., 2003; Narayanan and Johnston, 2007, 2008; Vaidya and Johnston, 2013; Das and Narayanan, 2014). Under pathologically induced or developmentally observed reduction in dendritic arborization, neurons lose their ability to process their inputs differentially based on their inputs, translating to errors in rate or temporal coding that are dependent on the presence of these functional maps (Magee, 2000; London and Hausser, 2005; Spruston, 2008; Wang, 2010; Narayanan and Johnston, 2012), unless concurrent homeostatic mechanisms are invoked to maintain functional map homeostasis in these neuronal structures (O'leary et al., 2014; Rathour and Narayanan, 2014). Therefore, future studies could focus on the role of dendritic atrophy in altering stratified input processing, and their implications for neural coding and homeostasis in hippocampal and cortical neuronal structures.

In addition to these, our results also outline the importance of dendritic morphology in the regulation of channelostasis in particular, and proteostasis in general. Specifically, it is well established that the localization, targeting and turnover of individual channel and protein molecules at specific dendritic locations in neurons with complex arborization is an extremely complex puzzle (Lai and Jan, 2006; Vacher et al., 2008; Nusser, 2012; Hanus and Schuman, 2013; Rathour and Narayanan, 2014). If dendritic morphology plays a critical role in the emergence of functional maps, it stands to reason that maintenance of homeostasis in these functional maps in the face of changes in dendritic arborization (baseline or pathologically or developmentally regulated changes) would have to follow different regimes of channelostasis for channels that mediate these functional maps. Thus, baseline or remodeling-induced variability in pyramidal neuron morphology needs to be systematically analyzed for their specific contributions to the proteostatic mechanisms behind functional map homeostasis. Additionally, the differences in pyramidal neuron morphology across the dorsal and ventral CA1 subregions are well established. Given this, it is important to address questions on the specific contributions of branching patterns in the observed differences in somatodendritic physiology and functional maps of dorsal vs. ventral CA1 pyramidal neurons (Dougherty et al., 2012, 2013; Marcelin et al., 2012a,b).

Finally, our results also pose the question on whether ion channel gradients are necessary and therefore express only in neurons with large dendritic arborizations. Specifically, let us consider that the premise for the presence of gradients in channel densities of active dendrites is to provide for somatodendritic normalization of certain physiological properties or to bestow location-dependent processing with reference to stratified incoming stimulus (Magee, 2000; Johnston and Narayanan, 2008; Sjöström et al., 2008; Spruston, 2008; Narayanan and Johnston, 2012; Nusser, 2012). Such normalization is necessary and stratified processing is feasible only in neurons that have large dendritic arborization, that translate to higher electrotonic lengths and compartmentalized influence fields of ion channels. However, if neurons possess minimal arborization and are electrotonically compact (Rall, 1977), the aforementioned purpose for the expression of channel gradients would be defeated. This is because several physiological properties would already be normalized owing to the compact structure, and gradients in ion channels would not translate to stratified processing of input stimulus as a result of constricted functional maps. Therefore, the analyses of proteostasis in complex dendrites (Lai and Jan, 2006; Vacher et al., 2008; Nusser, 2012; Hanus and Schuman, 2013; Rathour and Narayanan, 2014) should account for the morophological complexity and the electrotonic compactness of the neuronal structure (Vetter et al., 2001; Sjöström et al., 2008; Zhuchkova et al., 2013) in assessing the necessity for specific targeting of ion channels in achieving certain functional goals.

### Conflict of interest statement

The authors declare that the research was conducted in the absence of any commercial or financial relationships that could be construed as a potential conflict of interest.
